# Essential role of proteasomes in maintaining self-renewal in neural progenitor cells

**DOI:** 10.1038/srep19752

**Published:** 2016-01-25

**Authors:** Yunhe Zhao, Xueqin Liu, Zebin He, Xiaojie Niu, Weijun Shi, Jian M. Ding, Li Zhang, Tifei Yuan, Ang Li, Wulin Yang, Li Lu

**Affiliations:** 1 Department of Anatomy, Shanxi Medical University, Taiyuan, 030001, China; 2 Department of Physiology, East Carolina University Medical School, Greenville, 27834, USA; 3 Guangdong-Hong Kong-Macau Institute of CNS Regeneration, Jinan University, Guangzhou, 510632, China; 4 Guangdong Key Laboratory of Brain Function and Diseases, Jinan University, Guangzhou, 510632, China; 5 School of Psychology, Nanjing Normal University, Nanjing, 210097, China; 6 Center of Medical Physics and Technology, Hefei Institutes of Physical Science, Chinese Academy of Sciences, Hefei, 230031, China; 7 Cancer Hospital, Hefei Institutes of Physical Science, Chinese Academy of Sciences, Hefei, 230031, China

## Abstract

Protein turnover and homeostasis are regulated by the proteasomal system, which is critical for cell function and viability. Pluripotency of stem cells also relies on normal proteasomal activity that mitigates senescent phenotypes induced by intensive cell replications, as previously demonstrated in human bone marrow stromal cells. In this study, we investigated the role of proteasomes in self-renewal of neural progenitor cells (NPCs). Through both *in vivo* and *in vitro* analyses, we found that the expression of proteasomes was progressively decreased during aging. Likewise, proliferation and self-renewal of NPCs were also impaired in aged mice, suggesting that the down-regulation of proteasomes might be responsible for this senescent phenotype. Lowering proteasomal activity by loss-of-function manipulations mimicked the senescence of NPCs both *in vitro* and *in vivo*; conversely, enhancing proteasomal activity restored and improved self-renewal in aged NPCs. These results collectively indicate that proteasomes work as a key regulator in promoting self-renewal of NPCs. This potentially provides a promising therapeutic target for age-dependent neurodegenerative diseases.

The breakdown of protein homeostasis has been suggested to be tightly associated with the aging process^1^, because all cells have to keep a dynamic balance between protein synthesis and degradation in order to maintain their integrity and normal functions^2^. In fast-proliferating cells, it is particularly crucial to recycle obsolete macromolecules for *de novo* synthesis of subcellular compartments and molecules to satisfy the requirement of rapid proliferation and/or differentiation. Such a self-renewal ability of cells, however, is gradually compromised and eventually diminished with age^2^. Hallmarks of aged cells include increased accumulation of hyper-oxidative, misfolded, or abnormally-aggregated proteins, all of which result from the dysfunctional cell clearance mechanisms, especially the protein degradation pathway.

The proteasome-dependent degradation is one of such cellular clearance mechanisms for retaining intracellular protein homeostasis, which targets and subsequently degrades damaged, misfolded or redundant proteins^3^. The dysfunction of proteasomes, in turn, may contribute to the occurrence of many aging-related diseases^4^. A proteasome is a 2.5-MDa protein complex which comprises one 20S catalytic core subunit and one or two 19S regulatory subunits. Proteins destined for proteasome-mediated degradation are first subjected to ubiquitination, and then transported to proteasomes for further proteolysis^5^. The active proteolytic sites are located in the internal chamber of 20S core subunit, toward which protein substrates must enter through a narrow gate on the side of the core particle. The 19S subunit of proteasomes regulates the gate, whose conformational change further affect the overall proteolytic capacity.

Various studies have shown that proteasomal activity might be compromised during the aging process in both animals and cells, given that its decrease has been found in a variety of aged tissues in humans, non-human mammals, and even in lower organisms such as fruit flies^6^,^7^,^8^,^9^,^10^,^11^,^12^,^13^,^14^,^15^,^16^,^17^,^18^. Consistent observations have also been reported in aged organs including heart, spinal cord, muscle, liver and brain^2^. Impaired adipocyte differentiation, for example, was found in aged adipose tissues with the abnormal proteasomal structure and function^19^. The loss of function in the catalytic subunit PSMB8 leads to lipodystrophy^20^. Our previous studies have provided further evidence showing that inhibiting proteasomal activity results in senescence-like phenotypes in cultured human bone marrow stromal cells (hBMSCs)^18^,^21^. Although the age-dependent impairment of proteasomal activity has been found in various brain regions^2^,^3^, it still remains unclear whether this would have any impact on the activity or pluripotency of neural progenitor cells (NPCs). Here, we examined the expression and activity of proteasomes in the sub-ventricular zone (SVZ) of mouse brain tissues, as well as in the isolated NPCs from mice at different ages, in an attempt to elucidate the relationship between proteasomes and self-renewal of NPCs.

## Results

### Down-regulated expression of 20S proteasome in NPCs with aging

**Figure 1:**
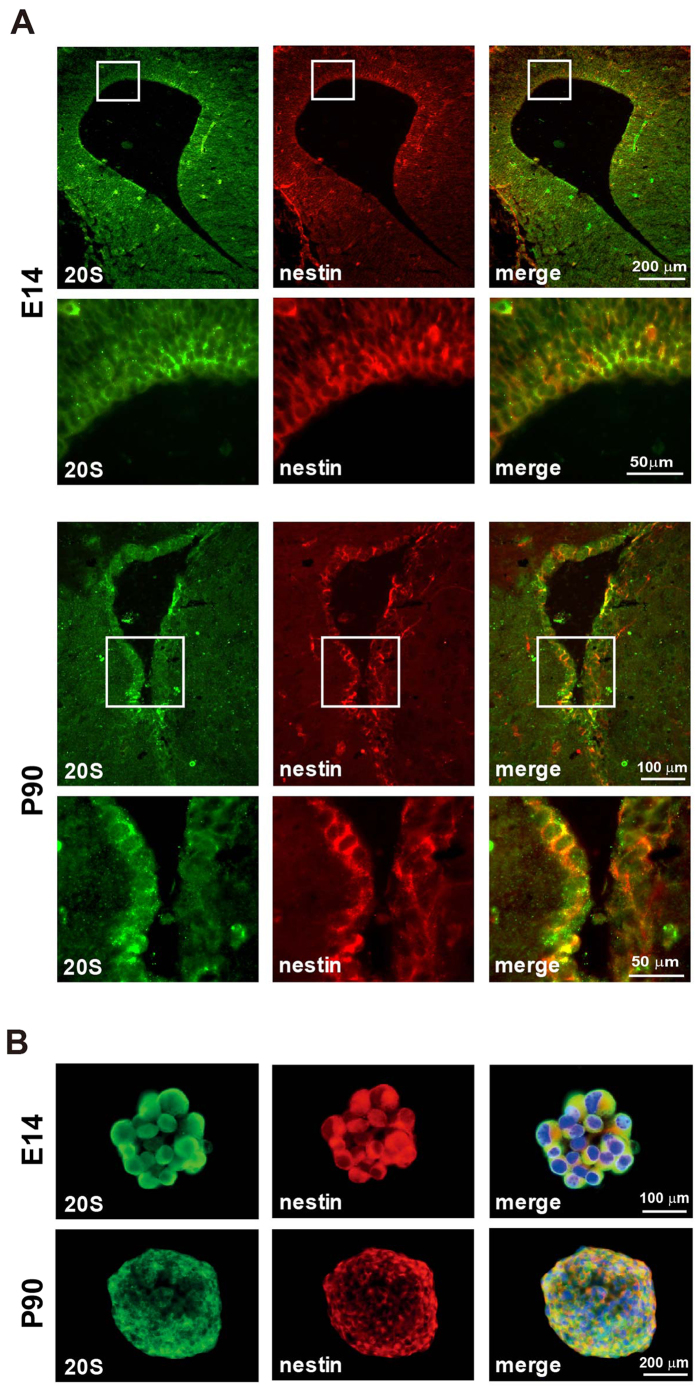
Expression of 20S proteasome in NPCs. (**A**) Representative images of coronal sections immunostained for 20S proteasome (Green) and nestin (Red) in the VZ/SVZ of E14 and the SVZ of P90 mice. Images with a higher magnification are presented. (**B**) Neurospheres from E14 and P90 mice were immunostained for 20S proteasome (Green) and nestin (Red), with nuclei counterstained by DAPI (Blue).

To explore the potential involvement of the proteasomal system in the aging process of NPCs, the expression profile of 20S proteasome was determined. As shown in Fig. 1A, the abundant co-localization between 20S proteasome and the neural progenitor marker nestin was observed in NPCs in the VZ/SVZ of E14 and the SVZ of P90 mice. Such a co-expression pattern was also found in cultured neurospheres of NPCs primarily isolated from the VZ/SVZ of E14 and the SVZ of P90 mice (Fig. 1B).

**Figure 2:**
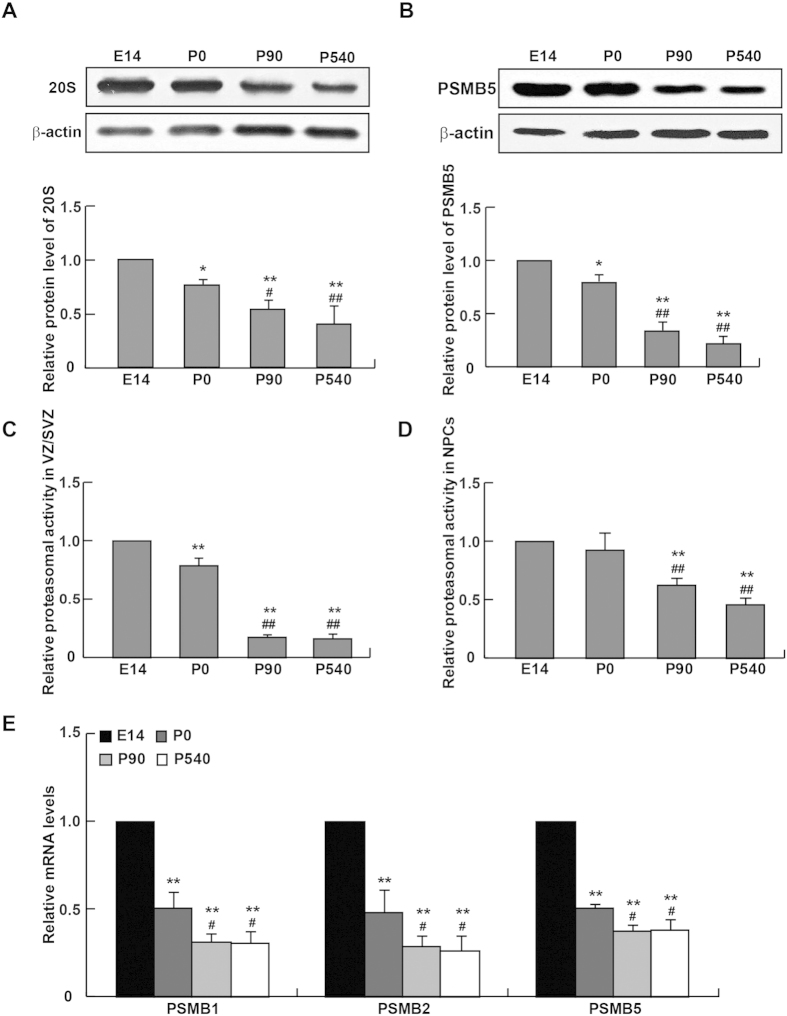
Down-regulation of proteasomes and their activity in aged NPCs. (**A,B**) Western-blotting analysis showed that the protein expressions of 20S proteasome and the subunit PSMB5 in the SVZ were decreased in P90 and P540 mice. (**C**) The proteasomal activity in the SVZ (or the VZ/SVZ at E14) was measured in mice at the time points specified. (**D**) The proteasomal activities in NPCs from P90 and P540 mice were significantly lower than that in E14 or P0 mice. (**E**) qPCR analysis on the expression of 20S proteasome subunits in NPCs isolated from E14, P0, P90 and P540 mice. *p < 0.05 and **p < 0.01 vs. E14, ^#^*p* < 0.05 and ^##^*p* < 0.01 vs. P0.

The quantitative analyses showed that, with age, protein levels of both 20S proteasome and its catalytic subunit PSMB5 were dramatically decreased (Fig. 2A,B). Consistently, both *in vivo* and *in vitro* experiments indicated remarkably-suppressed proteasomal activities in the SVZ homogenates and cultured NPCs from aged mice (Fig. 2C,D). Furthermore, by the qRT-PCR, key catalytic subunits of 20S proteasome, including PSMB1, PSMB2 and PSMB5, were unexceptionably down-regulated in NPCs derived from P90 and P540 mice (Fig. 2E). Collectively, these data showed a dynamic reduction in the expression of 20S proteasome with age. Considering the senescent phenotype of NPCs was detected in aged brains, it was reasonable to postulate that the impaired proteasomal function might be involved in this process.

### Compromised self-renewal capacity of NPCs in aged mice

**Figure 3:**
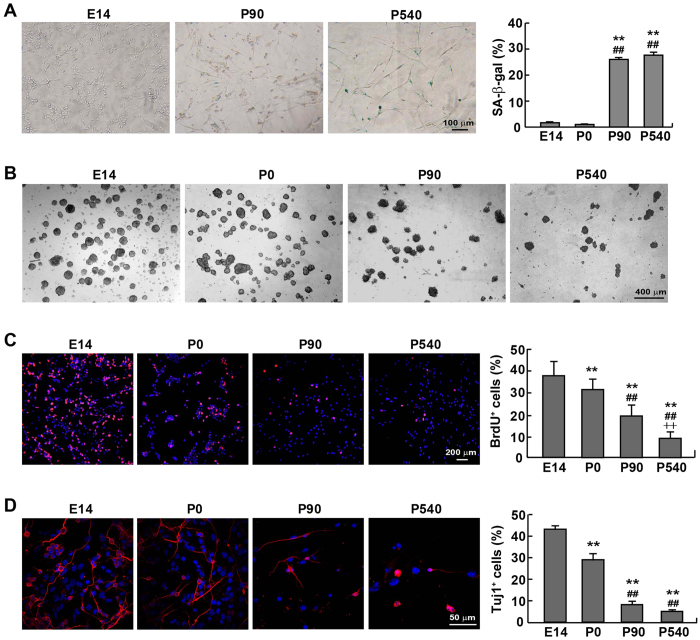
Defective proliferation and neuronal differentiation capacities in NPCs derived from P90 and P540 mice. (**A**) SA-β-gal activity was measured in NPCs isolated from E14, P0, P90 and P540 mice. The senescent cells were stained into blue. (**B**) Representative phase-contrast images of neurospheres from E14, P0, P90 and P540 mice in cultures. (**C**) Proliferation of NPCs was determined by BrdU incorporation. The percentage of dividing cells was gradually reduced with age. (**D**) After a 5-day induction for neuronal differentiation, NPCs from E14, P0, P90 and P540 mice were stained with the antibody against Tuj1, an early neuronal marker (Red). The neuronal differentiation potential was compromised in NPCs from P90 or P540 mice, compared with that from E14 and P0 mice. DAPI (blue) was used to counterstain nuclei. **p < 0.01 vs. E14, ^##^*p* < 0.01 vs. P0, ^++^*p* < 0.01 vs. P90.

Next, we compared the intrinsic properties of NPCs isolated from the SVZ (or the VZ/SVZ at E14) of mice at different ages. As displayed in Fig. 3A, the positive β-galactosidase signal was stronger in P90 and P450 NPCs than in their E14 counterparts. Concurrently, an age-dependent reduction of self-renewal was also supported by the result of the neurosphere formation assay (Fig. 3B). NPCs from E14 and P0 mice formed more spheres than those from aged mice. Further evidence by BrdU labeling demonstrated that the percentage of BrdU incorporation was gradually reduced with age (Fig. 3C), indicating a decreased proliferation in aged NPCs.

To directly assess the *in situ* proliferation capacity of NPCs, BrdU labeling was also conducted in mice at different ages, which showed that NPCs in the VZ/SVZ of E14 mice exhibited a substantially greater proliferation capacity than those in the SVZ of aged animals (Suppl. Fig. 1). In addition, neuronal differentiation was estimated by Tuj1 staining. The Tuj1^+^ cells were significantly reduced in NPCs from P90 and P540 mice, concurrent with an abnormal morphology (Fig. 3D). Together, our data suggested that the increasingly-aggravated senescent phenotype and progressive loss of pluripotency is age-dependent.

### Regulation of self-renewal in NPCs by proteasomal activity

**Figure 4:**
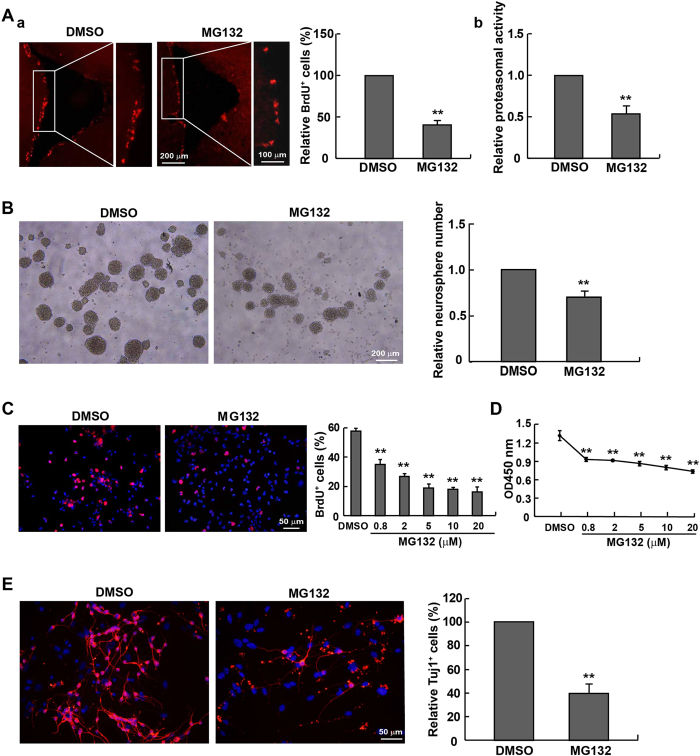
Appearance of senescence-like phenotypes in NPCs exposed to the proteasomal inhibitor MG132. (**A**) The number of proliferating cells in the SVZ was quantified by BrdU labeling 7 days after intraventricular injection with 10 μg MG132 or the vehicle DMSO. Notably, MG132 injection robustly inhibited NPCs proliferation *in vivo* (a), concurrent with a reduced proteasomal activity (b). (**B**) NPCs from E14 mice incubated with 0.2 μM MG132 for 24 hrs formed fewer neurospheres. (**C**) BrdU incorporation indicated a retarded proliferation of NPCs after an 8-hr treatment with MG132. The percentage of BrdU^+^ cells was reduced in a concentration-dependent manner. (**D**) After exposing NPCs to MG132 for 5 hrs, the cell viability was decreased in a concentration-dependent manner as measured by CCK-8 assay. (**E**) Tuj1 staining showed that treatment with MG132 attenuated the neuronal differentiation of NPCs *in vitro*. **p < 0.01 vs. DMSO control.

Both previous literatures and the results above confirmed the impaired proliferation and neuronal differentiation capacities in aged NPCs. We next tested whether proteasomal activity could directly affect the growth of NPCs. The proteasome inhibitor MG132 was injected into the mouse SVZ, followed by *in vivo* proliferation assay. The result of BrdU incorporation showed a decrease in proliferating cells following MG132 treatment (Fig. 4A). Similarly, MG132-treated NPCs (E14) formed fewer neurospheres *in vitro* than the DMSO vehicle controls (Fig. 4B). Moreover, proliferation of NPCs, as indicated by both BrdU incorporation (Fig. 4C) and CCK-8 assays (Fig. 4D), was inhibited in a concentration-dependent manner following addition of MG132. This somehow mimicked the decrease of SVZ proliferating cells during the aging process of mice which concurrently experienced a progressively-lowered proteasomal activity. Meanwhile, the *in vitro* neuronal differentiation of NPCs was also suppressed by MG132 (Fig. 4E). Interestingly, MG132 administration significantly raised intracellular levels of reactive oxygen species (ROS) (Suppl. Fig. 2A) and decreased mitochondrial membrane potential, an indication for impaired mitochondrial function (Suppl. Figs 2A and 3A,B). Hence, the age-dependent reduction of cell proliferation and neuronal differentiation may be attributed to the age-dependent decrease in proteasomal activity that may disrupt the endogenous regulation of oxidative stress and compromises the health of mitochondria.

**Figure 5:**
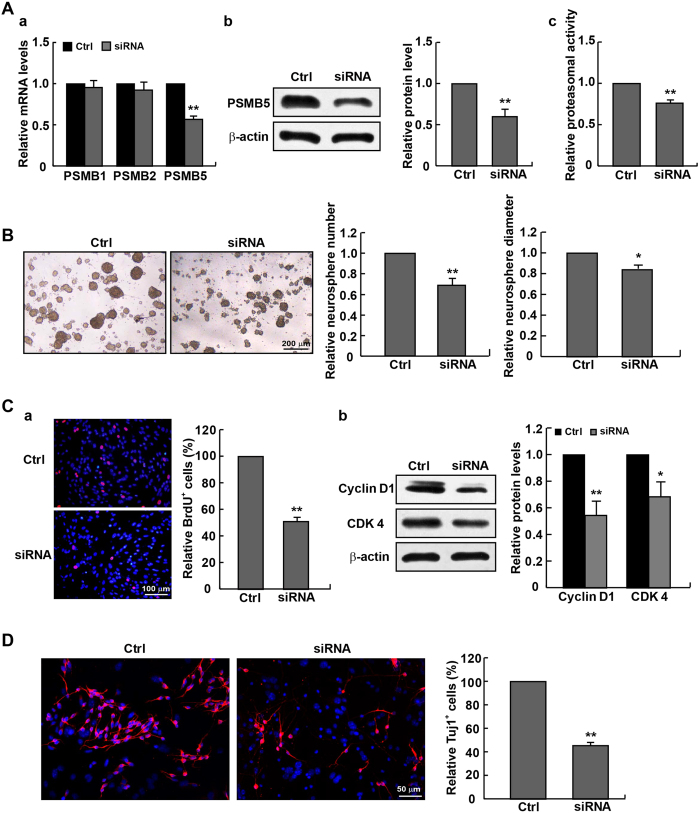
Suppressed proliferation and neuronal differentiation of cultured NPCs following PSMB5 knockdown-triggered decrease in proteasomal activity. (**A**) NPCs isolated from E14 mice were transfected with si-PSMB5 for 72 hrs. The knockdown efficiency was measured by qPCR (a) and western blotting (b). (c) PSMB5 knockdown reduced proteasomal activity in NPCs. (**B**) PSMB5 knockdown prevented the neurosphere formation, as indicated by decreases in both the number and diameter of neurospheres. (**C**) The decline of dividing cells was found in NPCs transfected with si-PSMB5 by BrdU incorporation (a). (b) Levels of the cell cycle-related proteins Cyclin D1 and CDK4 were lowered in NPCs treated with si-PSMB5. (**D**) NPCs transfected with either scramble siRNA or si-PSMB5 were cultured in the differentiation medium for 5 days. Neuronal differentiation was evaluated by the Tuj1/DAPI percentage. *p < 0.05 and **p < 0.01 vs. Scramble control.

We adopted another approach to examine the relationship between proteasomal activity and the pluripotency of NPCs, by means of manipulating the expression of PSMB5, the key catalytic subunit of 20S proteasome that decides the proteasomal activity^21^. The expression of PSMB5 was efficiently down-regulated after transfection with the specific siRNA pairs (Fig. 5A), accompanied by the inhibition of proteasomal activity, the predominant formation of smaller neurospheres (Fig. 5B), as well as the retarded NPCs proliferation evidenced by less incorporation of BrdU and down-regulation of cyclin D1 and CDK4 (Fig. 5C). Meanwhile, knocking down PSMB5 also caused an impaired capacity of neuronal differentiation in NPCs (Fig. 5D). Taken together, these data supported the potential correlation between the dysfunction of proteasomes and the impaired pluripotency of NPCs.

**Figure 6:**
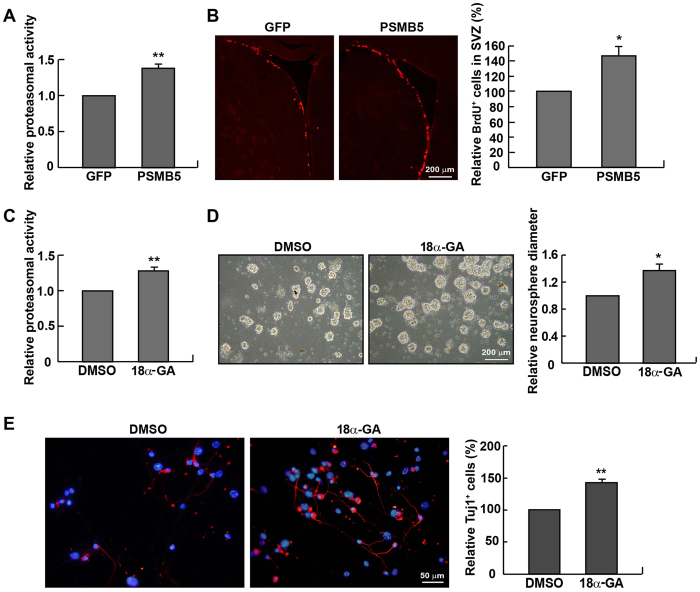
Rejuvenation of adult NPCs by up-regulating proteasomal activity. (**A**) Intraventricular injection with the recombinant PSMB5-overexpressing lentiviral particles for 7 days enhanced proteasomal activity in the SVZ of P90 mice. (**B**) PSMB5 over-expression increased BrdU^+^ cells in the SVZ (n = 6 mice). (**C**) NPCs derived from P90 mice were incubated with 18α-GA (2 μg/mL) for 10 days, followed by the proteasomal activity assay. (**D**) NPCs formed neurospheres with a larger size in the presence of 18α-GA. (**E**) After differentiation for 5 days, NPCs treated with 18α-GA generated more Tuj1^+^ cells than those treated with DMSO. *p < 0.05 vs. GFP or DMSO control, **p < 0.01 vs. GFP or DMSO control.

To further strengthen this conclusion, proteasomal activity was stimulated by either PSMB5 over-expression or the proteasome activator 18α-GA. Figure 6A displayed that the proteasomal activity was significantly raised in P90 mice administered with the recombinant lentiviral particles enabling the over-expression of PSMB5. As expected, the number of proliferating cells in the SVZ was also increased (Fig. 6B). Likewise, applying 18α-GA enhanced the proteasomal activity (Fig. 6C), enlarged the mean diameter of neurospheres (Fig. 6D), and improved neuronal differentiation (Fig. 6E). Moreover, treatment of 18α-GA lowered intracellular ROS levels (Suppl. Fig. 2B). At the same time, JC-1 staining revealed that mitochondrial function, evidenced by an increase in hyperpolarization of mitochondrial membranes, was enhanced in P90 NPCs treated with 18α-GA (Suppl. Fig. 3C). In sum, the pluripotency of NPCs relies, at least partly on proteasomal activity.

## Discussion

NPCs are characterized by their lifelong ability to self-renew and differentiate into various neural lineages in mammalian embryonic and adult brains. During development, these cells are potentially exposed to region- and time-specific stimuli, subsequently turning into functional neurons that constitute brain architecture^22^,^23^. The main neurogenic regions in adult mammals are the SVZ and the sub-granular zone of hippocampal dentate gyrus, where NPCs are continuously generated for neuronal replacement. However, with an unknown mechanism, both proliferation and neuronal differentiation capacities of NPCs isolated from the SVZ have been reported to gradually decrease in the process of aging^24^,^25^.

The present study has demonstrated that proteasomes may play a critical role in maintaining the pluripotency of NPCs. Proteasomes were found to be highly expressed by NPCs in the mouse VZ/SVZ, whose activity was progressively declined with age. Senescent NPCs with reduced proteasomal activity displayed impairments in proliferation *in vivo* and neurosphere formation *in vitro*, together with decreased neuronal differentiation. Inhibiting proteasomal activity by the inhibitor MG132 or by down-regulating the key catalytic subunit PSMB5 mimicked the aforementioned aging-induced phenotypes of NPCs; conversely, stimulating proteasomes by over-expressing PSMB5 or by the activator 18α-GA exerted the opposite effects. Therefore, proteasomes are likely involved in the regulation of the survival, maintenance and self-renewal of NPCs. Of note, NPCs are known for their temporal and regional heterogeneity, and the cellular composition and architecture in the SVZ are also dynamically changing throughout the life span^23^,^26^,^27^. Hence, proteasomal levels and activity in the isolated NPCs or SVZ tissue homogenates at different developmental stages could be potentially affected by the relative abundance and composition of NPC subtypes, besides aging *per se*. Elucidating this uncertainty in future requires the availability of more markers that are able to identify specific NPC subpopulations.

Cell aging is a complex process characterized by loss of normal functions that eventually leads to cell death^28^. One of the hallmarks in aging is the excess of super-oxidative, damaged intracellular macromolecules which disturb normal cellular functions and metabolism^29^. Accumulation of misfolded or damaged proteins during aging causes the interruption of cellular and tissue homeostasis. These perturbations potentially result in a variety of diseases including cancer, cardiovascular disease, diabetes, as well as some neurodegenerative disorders such as Alzheimer’s, Parkinson’s and Huntington’s diseases^30^,^31^. Proteasomes act as the regulatory hub in the proteostasis network, whose activity has been found to decline with age in various regions of the central nervous system, including hippocampus, cerebral cortex and spinal cord^32^,^33^,^34^. Although the exact mechanism mediating the reduction of proteasomal activity remains unclear, such a change has been speculated to compromise the physiological properties of neurons, oligodendrocytes and astrocytes, probably due to the accumulation of malfunctioned proteins that consequently disrupt neuronal cell functions such as neurotransmission, action transduction and axon guidance^35^. On the other hand, the current study also showed that proteasomes may directly affect the division of NPCs, both *in vivo* and *in vitro*. Concomitant with the insufficient clearance of aggregated/misfolded proteins, NPCs lost their pluripotency in proliferation and neuronal differentiation that permit the replacement of aged cells, thereby impairing the brain health. The dysregulation of self-renewal in NPCs can cause various brain disorders and cognitive deficits. Similar to other pluripotent stem cells, NPCs have the self-renewal capacity to retain their innate stem-cell property by self-clearance of macromolecules with oxidative-elicited insults and other damages. Prior work by us and others all indicated that enhancement of proteasomal activity could counteract the replicative senescence, which is beneficial to maintain the pluripotency of stem cells, such as the hBMSCs^18^,^21^.

This work offered the potential target for devising drugs against aging and neural degeneration, based on their association with the decrease in proteasomal activity. Given that the death of neurons as well as the senescence of NPCs may directly result from super-oxidation-triggered toxicity or secondarily-activated programmed cell death, recovering normal proteasomal activity would thereby benefit the clinical treatment of aging-related neural disorders by maintaining functional neurons or helping with *de novo* generation of neurons from NPCs. We previously increased proteasomal activity in hBMSCs by exogenously applying the proteasome activator 18α-GA or genetically over-expressing the β–subunit PSMB5^18^,^21^, and found that both methods could effectively improve cell integrity and ameliorate replicative senescence, in addition to enhancements of cell survival and neuronal differentiation following the brain transplantation of PSMB5-overexpressing hBMSCs. In the current study, we observed similar effects of 18α-GA on NPCs. Moreover, enhancing proteasomal activity with this agent in P90 NPCs also reduced intracellular ROS levels and restored the hyperpolarization of mitochondrial membranes. Conversely, administration of the proteasomal inhibitor MG132 raised ROS levels and lowered the mitochondrial membrane potential. Given that oxidative stress has been putatively regarded as a major contributor to cell senescence and excessive accumulation of intracellular ROS impairs the mitochondrial function^15^,^36^, improvement of proteasomal activity would be an effective way to rejuvenate senescent NPCs. Interestingly, certain natural extracts, such as oleuropein (a phenolic component from Olea *europaea*), have been found to strengthen the cell resistance to oxidative stress and thus extend lifespan by increasing proteasomal activity^37^. Other natural products, including pollens from bees^38^, lipid algae extracts^39^ and 2-hexyldecanol^40^ have proven to be proteasomal activators. The pharmaceutical properties of these natural products warrant further investigation, so as to select those potent proteasomal activators with minimal side effects.

In conclusion, our study provided both *in vivo* and *in vitro* evidence showing that proteasomal activity is an essential factor for self-renewal of NPCs, indicated by the correlation between aging-dependent impairment of proteasomal function and reduction of pluripotency. Promoting proteasomal activity can rejuvenate the senescent NPCs. The current work has underlined a critical role of proteasomes in maintaining the integrity of NPCs, which potentially offers a novel strategy to treat aging-related neurological diseases by stimulating proteasomal activity.

## Methods

### Animals

BALB/c mice at ages of embryonic day 14 (E14), postnatal day 0 (P0), postnatal day 90 (P90) and postnatal day 540 (P540) were used in this study. All experimental procedures have been pre-approved by Shanxi Animal Research Ethics Committee, and observed the available guidelines governing animal experiments.

### Neurosphere culture and differentiation

Neurospheres were generated from isolated NPCs in the VZ/SVZ of mouse embryos (E14) or the SVZ of postnatal mice (P0, P90 and P540) following well-established protocols^41^,^42^. Briefly, for culturing embryonic neurospheres, tissues micro-dissected from the dorsal forebrain of E14 mice were digested for 10 min at 37 °C in TrypLE Reagent (Invitrogen, Calsbad, CA). For culturing postnatal neurospheres, mouse brains were coronally sectioned, and the wall of lateral ventricles was obtained from sections of a coordinate between +0.5 mm and −1.5 mm relative to Bregma, followed by enzymatic dissociation in TrypLE Reagent supplemented with 20 U/mL papain (Worthington Biochemical Corp., Lakewood, NJ). Isolated cells were cultured at a density of 1 × 10^5 ^cells/mL in the DMEM-F12 proliferation medium containing B27 supplement (2%; Invitrogen), basic fibroblast growth factor (bFGF, 20 ng/mL; PeroTech, Rocky Hill, NJ), and epidermal growth factor (EGF, 20 ng/mL; PeroTech). The cultures were observed and photographed daily under a phase contrast microscope (Model CKX41, Olympus, Japan). Neurospheres were usually formed within 5 to 7 days, and those with diameters larger than 30 μm were later analyzed using the Image J software (NIH, Bethesda, MD).

For the differentiation assay, neurospheres were dissociated into single cells and re-suspended in the proliferation medium (2 × 10^5 ^cells/mL). Thereafter, NPCs were seeded onto the coverslips pre-coated with poly-L-ornithine (50 μg/mL, Sigma-Adrich, St Louis, MO) and laminin (10 μg/ml; Sigma-Aldrich). Following a 24-hr incubation, the differentiation medium containing 0.5% fetal bovine serum and 1% B27 supplement was replenished, and cells were induced to differentiate for 5 days, followed by fixation in 4% paraformaldehyde (PFA). The differentiated neuronal cells expressing the marker Tuj1 were immunophenotypically identified, as described below.

### MG132 and 18α-GA treatment

NPCs isolated from E14 mice were cultured in the proliferation medium added with the proteasome inhibitor MG132 (Beyotime Biotechnology, Jiangsu, China) at different concentrations for 5 hrs. For the differentiation assay, NPCs were exposed to MG132 (0.2 μM) for 5 days, with the culture medium changed every 24 hrs. NPCs isolated from P90 mice were treated with the proteasome activator 18α-GA (Sigma-Aldrich) at 2 μg/mL. Upon completion of treatments, cells were immunostained with antibodies against BrdU and Tuj1.

### Immunofluorescence detection

Immunofluorescence staining of tissue sections and cultured cells were performed as reported before^43^. Antibodies used in the current study included: rabbit anti-proteasome 20S α + β (Abcam, Cambridge, MA), rabbit anti-PSMB5 (Abcam), mouse anti-nestin (Millipore, Billerica, MA), mouse anti-BrdU (Abtech Biotechnology, Richmond, VA), mouse-anti Tuj1 (Covance, Richmond, CA), Alexa Fluor 555 goat anti-mouse IgG (Invitrogen) and Alexa Fluor 488 goat anti-rabbit IgG antibody (Invitrogen). Images were captured by a fluorescent microscope (Model BX51, Olympus, Japan), and analyzed by Image J software.

### Western blotting

Total proteins were extracted using the RIPA lysis buffer supplemented with the protease inhibitor cocktail (Thermo Fischer, Pittsburgh, PA). Protein concentrations were determined by a BCA Protein Assay kit (Beyotime). Equal amounts of proteins were separated by SDS-PAGE, and transferred to PVDF membranes (Millipore). Blots were incubated with the 5% milk for 1 hr at room temperature, and probed overnight at 4 °C with one of the following primary antibodies: rabbit anti-proteasome 20S α + β (Abcam), rabbit anti-PSMB5 (Abcam), rabbit anti-CDK4 (Abtech Biotechnology), rabbit anti-CyclinD1 (Epitomics, Burlingame, CA) and mouse-anti β-actin (Sigma-Aldrich). On the next day, membranes were thoroughly rinsed in the phosphate-buffer saline (PBS) containing 0.1% Tween-20 (PBST), and incubated with horseradish peroxidase-conjugated goat anti-rabbit IgG or goat anti-mouse IgG secondary antibodies for 1 hr. Bands were then visualized by the ECL detection kit (GE Healthcare Life Science, Pittsburgh, PA), and documented on films. Intensities of bands were analyzed by densitometry using the QuantityOne software (Bio-Rad, Hercules, CA).

### RNA isolation and the quantitative real-time PCR

Total RNA was extracted with the TRIzol reagent (Invitrogen), and reverse-transcribed into cDNA templates using the PrimeScriptTM RT reagent kit (Takara, Japan) following the manufacturer’s instruction. Gene expression levels were quantified using the Maxima SYBR Green qPCR Master Mix (Takara) on the StepOne Plus Real-Time PCR System (Applied Biosystems Inc., Foster City, CA). Sequences of primers were listed in Supplementary Table 1.

### BrdU incorporation and quantitative analysis

To assess the proliferation of NPCs in the SVZ, mice were intraperitoneally injected with BrdU (30 mg/kg body weight) for six times at 2-hr intervals, and sacrificed 24 hrs later. Serial coronal sections with a thickness of 16 μm were sliced from the most rostral site of the corpus callosum to the third ventricle (crossing of the anterior commissure). Every fourth specimen was collected and mounted on glass slide, followed by the immunodectection of incorporated BrdU, as described before^44^. For each mouse, 10 sections were counted to calculate the total number of BrdU^+^ cells in the SVZ.

The *in vitro* proliferation assay for NPCs grown on coverslips was performed as previously reported^18^. Briefly, NPCs were maintained in the proliferation medium, and BrdU was added to a final concentration of 10 μM during the last 7 hours of culture. Samples received the BrdU immunofluorescence detection as detailed above. For each coverslip, BrdU^+^ cells in 10 different non-overlapping fields under 20-fold magnification were quantified.

### Senescence-associated β-galactosidase staining

Replicative senescence was evaluated by β-galactosidase staining as previously described^18^,^21^. In brief, the intracellular activity of pH-dependent senescence-associated β-galactosidase (SA-β-gal) was examined with SA-β-gal Staining Kit (Beyotime) following the suggested protocol. Senescent cells stained into blue color were counted under 20-fold magnification in a double-blind manner. For each sample, 10 different non-overlapping fields were randomly selected, and the percentage of senescent cells was calculated.

### Intracerebroventricular injection of MG132

P90 mice anaesthetized by intraperitoneal injection of ketamine (90 mg/kg body weight) and xylazine (10 mg/kg body weight) were fixed on a stereotactic apparatus. Mice were intracerebroventrically injected with 1 μl MG132 (21 mM in 10% DMSO) or vehicle (10% DMSO) using a Hamilton syringe (Hamilton Company, Reno, NV). The coordinates used were (relative to Bregma): posterior, 0.2 mm; lateral, 1.0 mm; and ventral, 2.0 mm. Seven days after injection, mice were sacrificed for the BrdU incorporation assay as described above.

### Lentiviral infection in the SVZ

The generation of recombinant PSMB5-overexpressing lentiviral particles was described before^21^,^45^. The titer of viral stocks was adjusted to 1 × 10^8^ colony-forming units (cpu) per mL. A 2-μl viral suspension containing polybrene (4 μg/mL; Sigma-Aldrich) was injected into the SVZ [Coordinates (relative to Bregma): anterior, 0.6 mm; lateral, 1.6 mm; and ventral, from 3.4 towards 1.8 mm]. After injection, the needle was retained inside the cranial cavity for 5 min before slowly withdrawn. To label proliferating cells, BrdU injections were performed 7 days after the surgery.

### Proteasomal activity assay

As recorded before^21^, protein homogenates were prepared and chymotrypsin-like proteasomal activity was measured with the 20S Proteasome Activity Assay Kit (Millipore). Briefly, equal amounts of protein lysates were incubated with the LLVY-AMC substrate for 1 hr at 37 °C, and the intensity of free AMC fluorescence was quantified using a 380/460 nm filter set in a SpectraMax fluorescence microplate reader (Molecular Devices, US).

### RNA interference

The siRNA targeting PSMB5 (5′-GCACCAUGAUCUGUGGCUGGGAUAA-3′) was synthesized by Genepharma Corp (Shanghai, China). Gene knockdown efficiency was determined by qPCR and Western blotting. The transfection of siRNA and scramble RNA control (4 μM) was conducted on NPCs from E14 mice with the Nucleofector II system (Lonza, Walkersville, MD).

### CCK-8 assay

To evaluate cell viability, CCK-8 kits (Dojindo Laboratories, Japan) were utilized following the manufacturer’s instruction. In brief, neurospheres from E14 mice were dissociated into single cells and plated into 96-well plate (6 × 10^4 ^cells/well). NPCs were recovered for 24 hours before addition of MG132 (final concentrations: 0.8 ~ 20 μM) for a 5-hr incubation. The CCK-8 assay solution was added to each well during the last 4 hours of culture. Absorbance at 450 nm was obtained with a microplate reader (Multiskan GO, Thermo Scientific).

### Reactive oxygen species detection assay

Intracellular accumulation of reactive oxygen species (ROS) was measured as previously described^21^. Briefly, NPCs cultured in 96-well plates were pretreated with MG132 or 18α-GA as specified, using DMSO vehicle of the same percentage as the control. After washed twice with PBS, cells were incubated with the medium containing 10 μM DCFH-DA for 1 hr at 37 °C. Fluorescence was monitored by a microplate fluorometer (SpectraMax, Molecular Devices) at excitation and emission wavelengths of 485 and 535 nm, respectively.

### Mitochondrial membrane potential assay

As previously documented, the mitochondrial membrane potential in NPCs was monitored by JC-1 dye (Beyotime)^46^. In brief, NPCs treated with MG132 or 18α-GA were bathed in the working solution containing JC-1 (5 μg/ml) for 20 min at 37 °C. Thereafter, cells were viewed under a confocal microscopy equipped with an incubation system (DetalVision, Applied Precision). For each sample, images of at least 10 individual fields were captured and analyzed with MetaMorph software (Sunnyvale, CA). Fluorescence intensities at 590 nm (red, indicating hyperpolarization) and 530 nm (green, indicating depolarization) were obtained, and the red/green ratio was applied to estimate the health of mitochondria^47^.

### Statistical analysis

All experiments were performed at least in triplicates. Comparisons were accomplished by one-way analysis of variance (ANOVA) with LSD post-hoc test or student’s *t*-test, as appropriate, using the SPSS software (Chicago, IL). Data in all figures were presented as mean ± standard errors of the mean (SEM). A statistical significance was defined as *p* < 0.05.

## Additional Information

**How to cite this article**: Zhao, Y. *et al.* Essential role of proteasomes in maintaining self-renewal in neural progenitor cells. *Sci. Rep.* **6**, 19752; doi: 10.1038/srep19752 (2016).

## Supplementary Material

Supplementary Information
