# Supplementary material for: Essential role of proteasomes in maintaining self-renewal in neural progenitor cells
Source: Sci Rep. 2016 Jan 25;6:19752. doi: 10.1038/srep19752 (PMC4726439; doi:10.1038/srep19752)

***Supplementary Information***

**Essential role of proteasomes in maintaining self-renewal in neural progenitor cells**

Yunhe Zhao^1,#^, Xueqin Liu^1,#^, Zebin He^1,#^, Xiaojie Niu^1,#^, Weijun Shi^1^, Jian M. Ding^2^, Li Zhang^3,4^, Tifei Yuan^5^, Ang Li^3,4^,*, Wulin Yang^6^,*, Li Lu^1^,*

^1^Department of Anatomy, Shanxi Medical University, Taiyuan, 030001, China

^2^Department of Physiology, East Carolina University Medical School, Greenville, 27834, USA

^3^Guangdong-Hong Kong-Macau Institute of CNS Regeneration, Jinan University, Guangzhou, 510632, China

^4^Guangdong Key Laboratory of Brain Function and Diseases, Jinan University, Guangzhou, 510632, China

^5^School of Psychology, Nanjing Normal University, Nanjing, 210097, China

^6^Center of Medical Physics and Technology, Hefei Institutes of Physical Science, CAS, Hefei, 230031, China

^#^These authors contributed equally to this work.

*Correspondence to: Li Lu, Ph.D, Department of Anatomy, Shanxi Medical University, Taiyuan 030001, China, Tel.: +86-351-4135787, Fax: +86-351-2022548, E-mail: [luli7300@126.com](mailto:luli7300@126.com); or Wulin Yang ([yangw@cmpt.ac.cn](mailto:yangw@cmpt.ac.cn)), or Ang Li ([anglijnu@jnu.edu.cn](mailto:anglijnu@jnu.edu.cn)).

**Supplementary Table 1. Gene-specific Primers for Real-time PCR**

| **Target** | **Forward Primer** | **Reverse Primer** |
| --- | --- | --- |
| PSMB1 | TAATTGGCTGCAGTGGTTTCC | AAGCGCCGTGAGTACAGGAT |
| PSMB2 | GATGAAGGACGATCATGACAAGAT | TGGGAGACAATTCATATCCATTCC |
| PSMB5 | CGCAGCAGCCTCCAAACT | GAAGGCGGTCCCAGAGATC |
| GAPDH | TGTGTCCGTCGTGGATCTGA | TTGCTGTTGAAGTCGCAGGAG |

**Supplementary Figure Legends**

**Suppl. Figure 1. Age-dependent reduction of the number of proliferating cells in the mouse SVZ**

Two hours after the intraperitoneal injection with BrdU (100 mg/kg body weight), mice at different ages were sacrificed, with their brains coronally sectioned and immunostained for identifying the proliferating cells in the SVZ (or the VZ/SVZ for E14 mice). The quantitative result confirmed that the number of BrdU^+^ cells was progressively reduced during the aging process of mice. ***p* < 0.01 vs. E14, ^##^*p* <0.01 vs. P0, ^++^*p* < 0.01 vs. P90.

**Suppl. Figure 2. Changes of ROS levels in NPCs following MG132 and 18α-GA treatments**

**A**, Intracellular levels of ROS were quantified by DCF fluorescence. Incubating E14 NPCs with the proteasome inhibitor MG132 for 5 hrs raised the ROS level in a concentration-dependent manner. **B**, Compared with DMSO control, treating P90 NPCs with the proteasome activator 18α-GA (2 μg/mL) lowered the ROS level. **p* < 0.05 and ***p* < 0.01 vs. DMSO control.

**Suppl. Figure 3. Effects of MG132 and 18α-GA on the mitochondrial membrane potential of NPCs**

**A**, Representative photos of JC-1 staining. Red fluorescence emitted by the polymeric form of JC-1 is associated with membrane hyperpolarization that is a characteristic of healthy mitochondria, whereas green fluorescence by the monomeric form indicates mitochondrial membrane depolarization. Nuclei were counterstained with Hoechst 33342 (blue). **B**, The depolarization of NPCs mitochondrial membranes was quantified by the red/green fluorescence intensity ratio. The bar chart concludes the results in E14 NPCs treated with MG132 at the concentrations specified. **C**, The intensity ratio of red/green fluorescence was significantly increased in P90 NPCs treated with 18α-GA (2 μg/mL). ***p* < 0.01 vs. DMSO control.


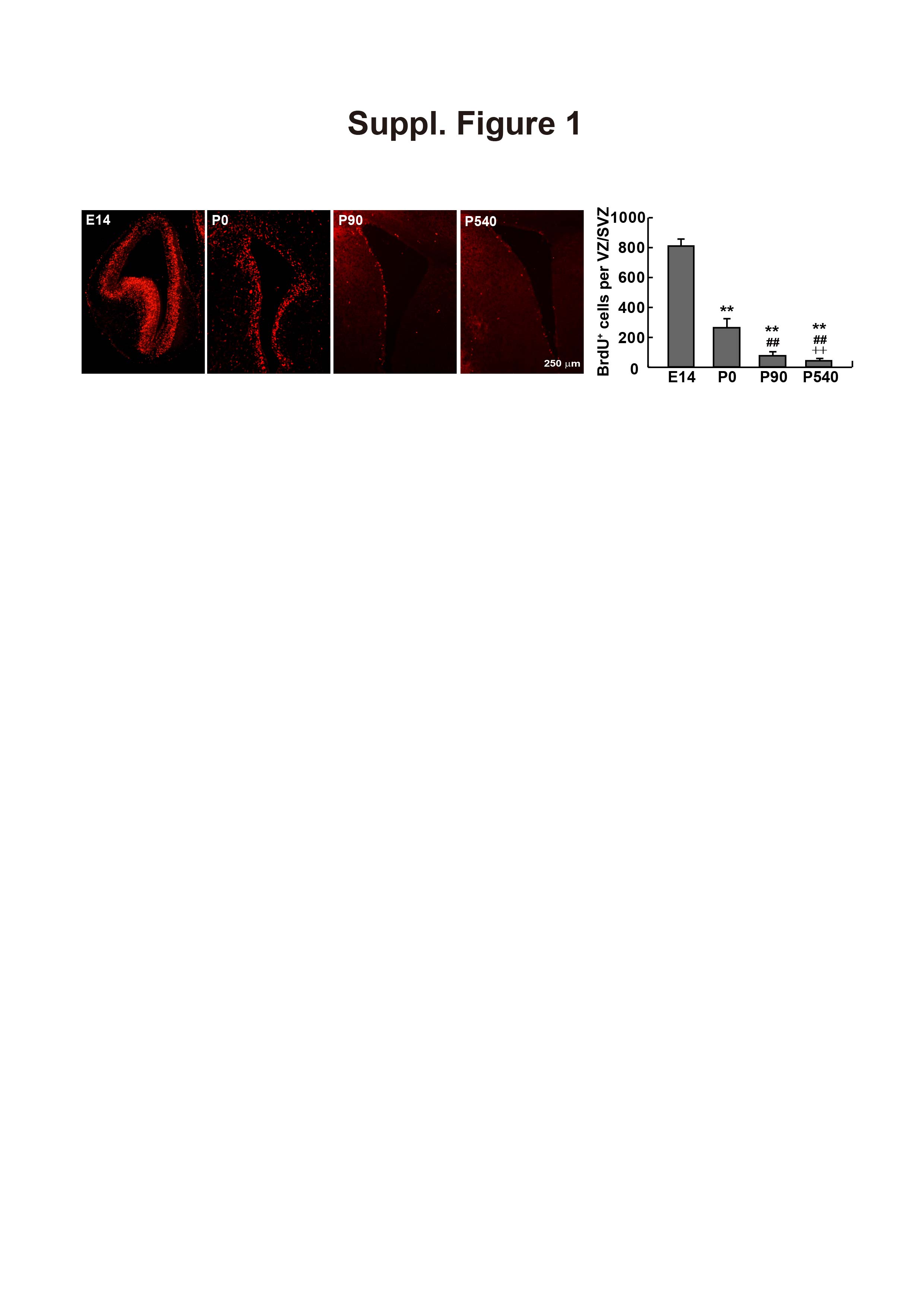


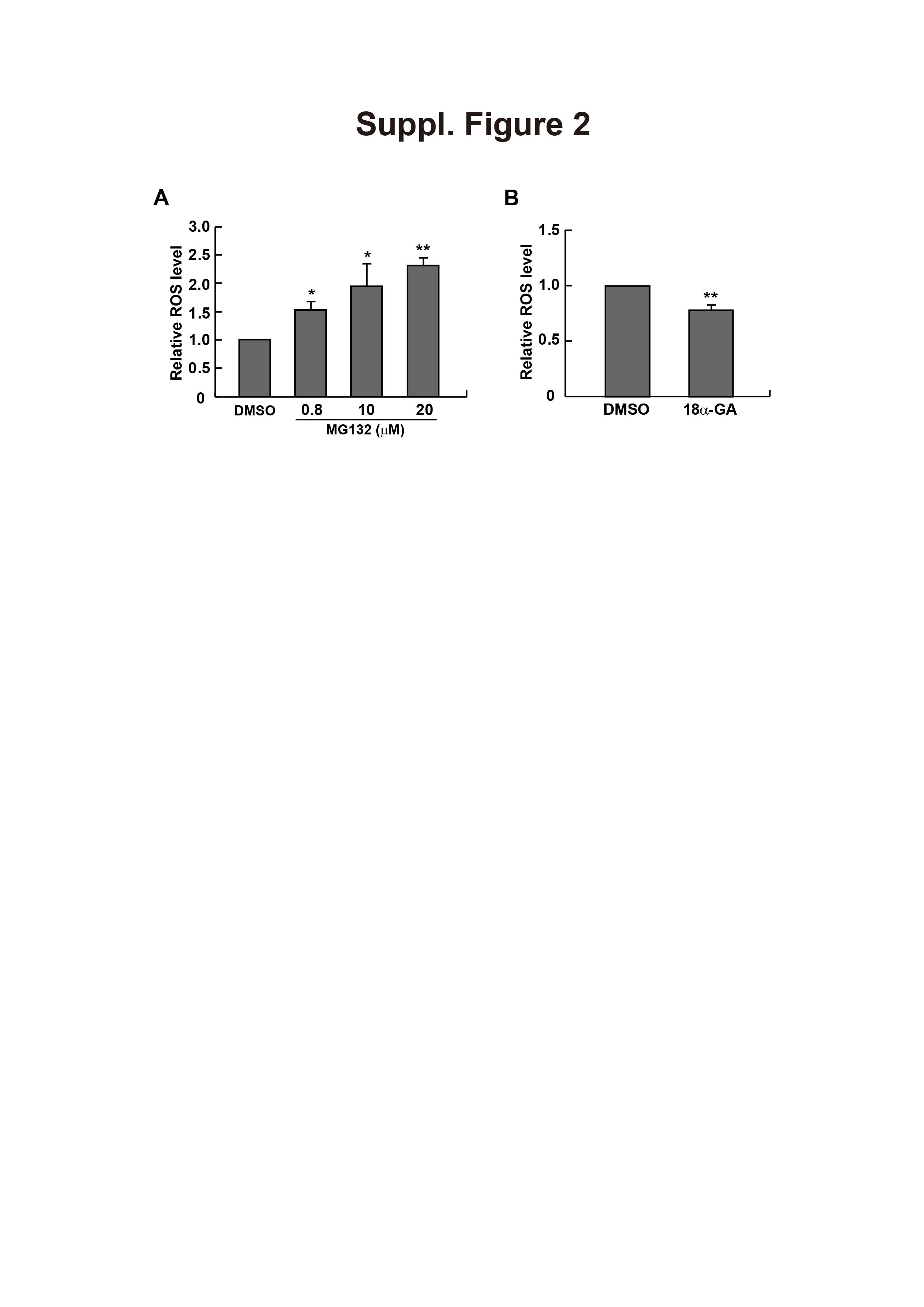


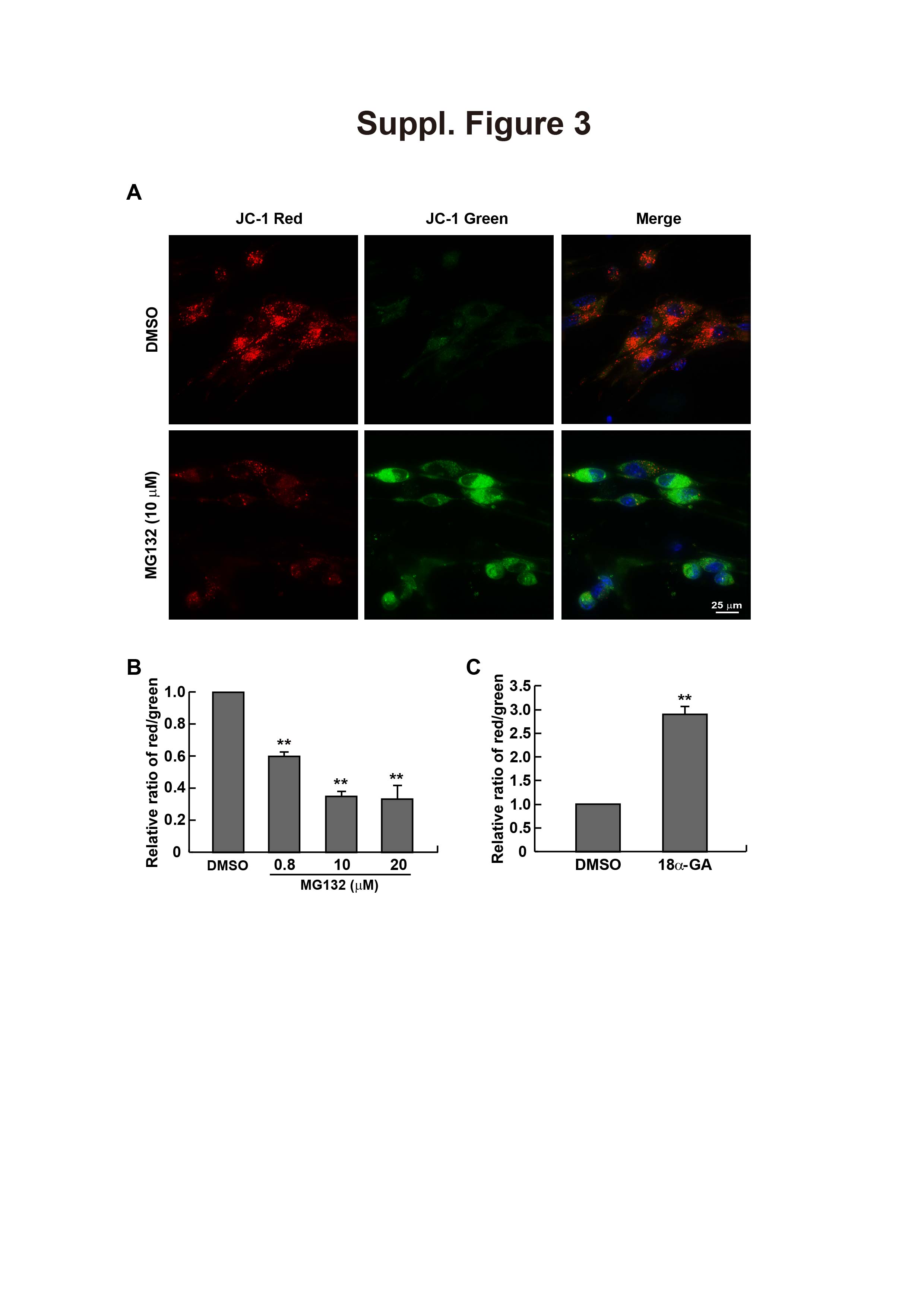

Supplement: Supplementary Information [file srep19752-s1.doc]
